# Novel rat model of multiple mitochondrial dysfunction syndromes (MMDS) complicated with cardiomyopathy

**DOI:** 10.1002/ame2.12193

**Published:** 2021-12-06

**Authors:** Yahao Ling, Jiaxin Ma, Xiaolong Qi, Xu Zhang, Qi Kong, Feifei Guan, Wei Dong, Wei Chen, Shan Gao, Xiang Gao, Shuo Pan, Yuanwu Ma, Dan Lu, Lianfeng Zhang

**Affiliations:** ^1^ Key Laboratory of Human Disease Comparative Medicine National Health Commission of China (NHC) Institute of Laboratory Animal Science Peking Union Medical College Chinese Academy of Medical Sciences Beijing China; ^2^ Beijing Engineering Research Center for Experimental Animal Models of Human Diseases Institute of Laboratory Animal Science Peking Union Medical College Chinese Academy of Medical Sciences Beijing China

**Keywords:** cardiomyopathy, energy metabolism, ISCA1, multiple mitochondrial dysfunction syndromes (MMDS), rat model

## Abstract

**Background:**

Multiple mitochondrial dysfunction syndromes (MMDS) presents as complex mitochondrial damage, thus impairing a variety of metabolic pathways. Heart dysplasia has been reported in MMDS patients; however, the specific clinical symptoms and pathogenesis remain unclear. More urgently, there is a lack of an animal model to aid research. Therefore, we selected a reported MMDS causal gene, *Isca1*, and established an animal model of MMDS complicated with cardiac dysplasia.

**Methods:**

The myocardium‐specific *Isca1* knockout heterozygote (*Isca1* HET) rat was obtained by crossing the *Isca1* conditional knockout (*Isca1* cKO) rat with the *α myosin heavy chain Cre* (*α‐MHC‐Cre*) rat. Cardiac development characteristics were determined by ECG, blood pressure measurement, echocardiography and histopathological analysis. The responsiveness to pathological stimuli were observed through adriamycin treatment. Mitochondria and metabolism disorder were determined by activity analysis of mitochondrial respiratory chain complex and ATP production in myocardium.

**Results:**

ISCA1 expression in myocardium exhibited a semizygous effect. *Isca1* HET rats exhibited dilated cardiomyopathy characteristics, including thin‐walled ventricles, larger chambers, cardiac dysfunction and myocardium fibrosis. Downregulated ISCA1 led to deteriorating cardiac pathological processes at the global and organizational levels. Meanwhile, HET rats exhibited typical MMDS characteristics, including damaged mitochondrial morphology and enzyme activity for mitochondrial respiratory chain complexes Ⅰ, Ⅱ and Ⅳ, and impaired ATP production.

**Conclusion:**

We have established a rat model of MMDS complicated with cardiomyopathy, it can also be used as model of myocardial energy metabolism dysfunction and mitochondrial cardiomyopathy. This model can be applied to the study of the mechanism of energy metabolism in cardiovascular diseases, as well as research and development of drugs.

## 1 INTRODUCTION

Multiple mitochondrial dysfunction syndromes (MMDS) presents as impaired mitochondrial metabolism and energy production, which can ultimately lead to damage to both the structure and function of mitochondrial complex, and thus the function of mitochondrial respiratory complex. MMDS is accompanied by serious damage to various metabolic pathways, resulting in conditions such as mitochondrial encephalopathy, myopathy and respiratory insufficiency.^1^, ^2^, ^3^, ^4^ Energy generation impairment is considered as the pathogenic basis of MMDS. It has been reported that mutations in genes involved in synthesis of ISC may seriously damage mitochondrial metabolic processes and thus ultimately disrupt energy production.^3^, ^5^, ^6^ Mutated genes that have been reported include BOLA3, IBA57, ISCA1 and PMPCB.^2^, ^3^, ^5^, ^6^, ^7^, ^8^

Abnormal muscle or heart development has been reported in patients with MMDS. BOLA3 deficient patients developed cardiomyopathy,^9^ and IBA57 deficient patient developed severe myopathy.^10^ ISCA1 deficiency results in spasticity with exaggerated deep tendon reflexes,^7^, ^11^, ^12^ while PMPCB deficient patients develop dystonia.^8^ The human heart is a very energy‐intensive tissue and mitochondrial oxidative phosphorylation is responsible for almost all of the ATP production (>95%) in adult mammalian hearts.^13^, ^14^ Therefore, maintaining mitochondrial function is essential for the functioning of the cardiac pump.

However, the specific mechanism by which MMDS induces cardiac developmental disorders is not yet well understood, and there is an urgent need for suitable *in vivo* animal models to aid research.

**FIGURE 1:**
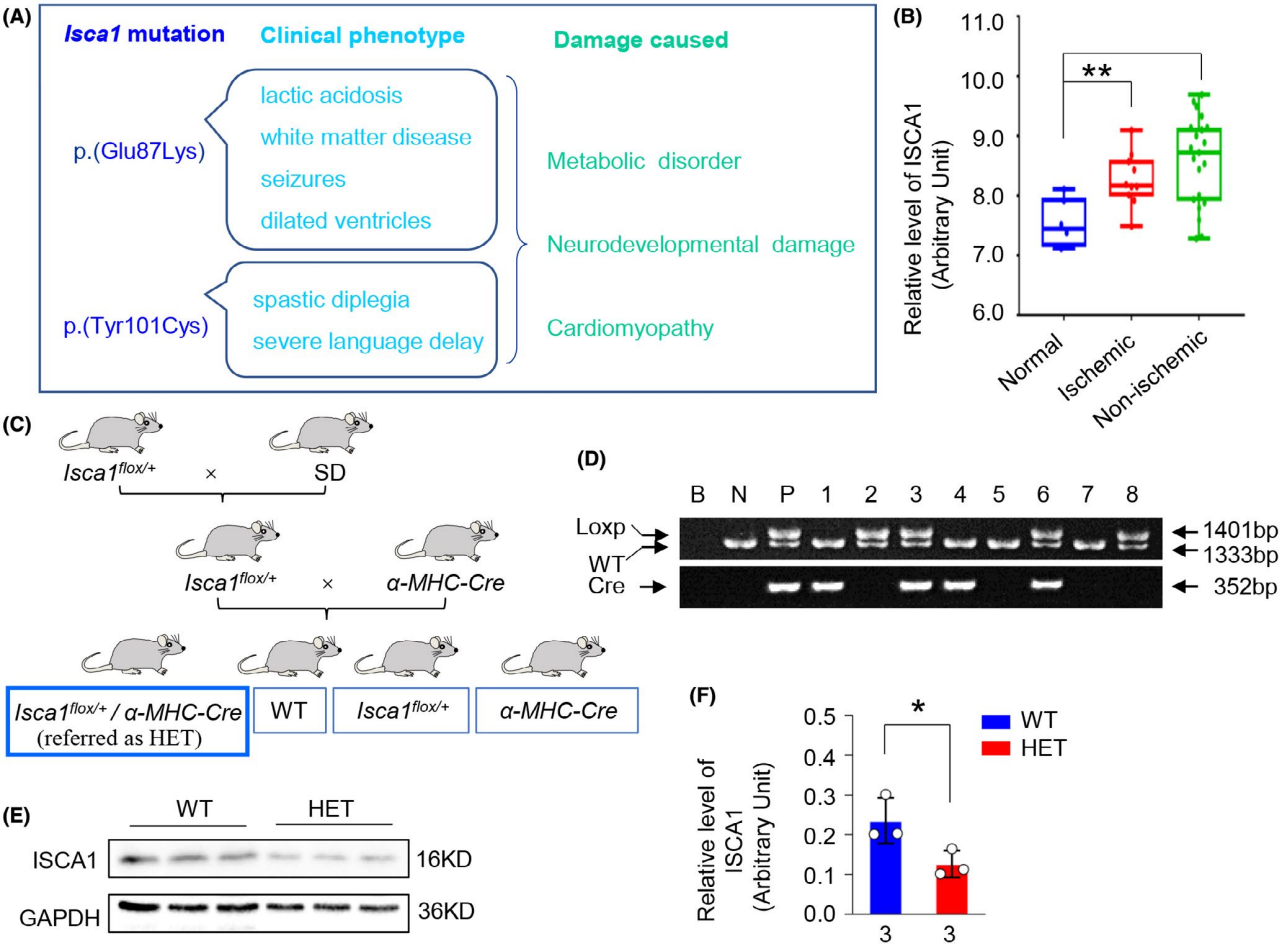
The expression of ISCA1 under cardiomyopathy and establishment of myocardium specific *Isca1* knockout heterozygote (HET) rat. (A) Phenotypic characteristics of clinical patients with Isca1 mutation. (B) ISCA1 expression in myocardium from ischemic and non‐ischemic cardiomyopathy patients compared with normal donors (*p* < .01, data from GSE1869, *n* = 6 in normal group; *n* = 10 in ischemic group; *n* = 21 in non‐ischemic group). (C) The HET (*Isca1^flox/+^/α‐MHC‐Cre*) rat was obtained by crossing *Isca1*‐floxed rats with *α‐MHC‐Cre* rats. Genotyping assays were performed using PCR (D) and *Isca1* expression in the myocardium of HET rats was detected using western blot (E–F, *n* = 3 rats per group, *p < .05, vs. WT)

ISCA1 is the A‐type ISC protein, which is part of the mitochondrial [4Fe–4S] cluster assembly machinery of key respiratory enzymes.^15^ Two variants have been reported, p.(Glu87Lys) and p.(Tyr101Cys), and ISCA1‐related MMDS results mainly in metabolic disorders, neurodevelopmental damage and cardiomyopathy,^7^, ^11^, ^12^, ^15^ as summarized in Figure 1A.

We next analyzed the expression of *Isca1* within the GSE1869 dataset from the Gene Expression Omnibus (GEO, https://www.ncbi.nlm.nih.gov/geo), and we found that it increased significantly in hearts with both ischemic cardiomyopathy and non‐ischemic cardiomyopathy with end‐stage (Figure 1B). Those findings suggested to us that abnormal ISCA1 expression may affect the development of the heart, and thus an animal model tool of MMDS complicated with cardiac dysphasia could be established by modifying the expression of the ISCA1 gene.

In this study, a myocardium specific *Isca1* knockout heterozygote rat model was established that exhibited the typical MMDS pathological phenotype of dilated cardiomyopathy (DCM) and complex mitochondrial damage to both the structure and function of the myocardium. This rat model can be used to investigate energy metabolism and in research and development of drugs for cardiovascular and metabolic diseases.

## 2 METHODS

### 2.1 Generation of myocardium specific *Isca1* knockout rats

The *Isca1*‐floxed rats (referred to as *Isca1* conditional knockout, *Isca1* cKO) were produced using the CRISPR/Cas9 system.^16^ The rats produced were confirmed as *Isca1* cKO by sequencing and genotyping with specific primers (Table S1). *α‐MHC‐Cre* transgenic rats were established in our laboratory as previously reported.^17^, ^18^ Animals carrying the *α‐MHC‐Cre* gene were identified using PCR analysis of tissue genomic DNA using Cre‐specific primers (Table S1). Myocardium specific *Isca1* knockout heterozygote rats were generated using the *Cre‐lox*P system by crossing *Isca1* cKO rats with *α‐MHC‐Cre* rats. Offspring with the genotype of positive *α‐MHC‐Cre* transgene and *Isca1^flox/+^* were selected as myocardium specific *Isca1* knockout heterozygote (*Isca1^flox/+^/α‐MHC‐Cre*) rats and are referred to as *Isca1* HET rats in this manuscript (Figure 1C). The *Isca1* HET rats were used in subsequent analyses and WT littermates were used as controls. Further information on the rats used in this study can be found in our rat database (www.ratresource.com).

The background strain of the rats used in this study was the Sprague‐Dawley rat. All rats were maintained in standard cages in an Association for Assessment and Accreditation of Laboratory Animal Care (AAALAC) accredited animal facility. All animal experiments were approved by the Animal Care and Use Committee of the Institute of Laboratory Animal Science of Peking Union Medical College (Permission No. MYW20002).

### 2.2 Genomic DNA preparation and genotyping

The EasyPure^®^ Genomic DNA Kit (China, Trans Gen Biotech, EE101‐22) was used to extract genomic DNA from the tail tissue of 7‐day‐old rats according to the manufacturer's instructions. The rat tail tissue was disaggregated by incubating the tissue in a lysis buffer with proteinase K (20 mg/ml) in a swing bed at 55°C for 6–8 h. A silica‐based column specifically for binding DNA was used to separate the genomic DNA from the tissue lysate. The genomic DNA was eluted by adding 150 μl of elution buffer.^17^, ^18^

### 2.3 Measurement of basic physiological parameters

Blood pressure (BP) was measured by the tail‐cuff method (Visitech Systems, BP‐2000, USA) in conscious rats (3 times per week). Weekly BP data are the average of three measurements per week taken at 10:00 a.m. For electrocardiography determination, the rats were first anesthetized with isoflurane (1.5%–2.5%) and then held in a supine position, using a heating pad to maintain body temperature. The limb leads were place subcutaneously with lead II derivation. Traces were recorded using BIOPAC system (MP150, USA) and analyzed using the Acqknowledge software (USA).^19^

### 2.4 Survival analysis

The cumulative death rates of the WT littermate control rats and the *Isca1* HET rats (*Isca1^flox/+^/α‐MHC‐Cre*) were calculated from birth to 18 months of age. A pathologist performed the autopsy after the death of each rat and pathological changes and morphological alterations of the heart were recorded. The Kaplan–Meier curves were compared using the log‐rank test (GraphPad Prism8 software).^20^

### 2.5 Adriamycin treatment

Two‐month‐old rats were used for Adriamycin (ADR) treatment. ADR was injected intraperitoneally in a constant volume of saline at 2.5 mg/kg every other day for a total of 2 weeks as previously reported.^21^, ^22^ The saline groups received the same volume of saline. Echocardiography was performed on all rats on day 0 (the day before ADR treatment). All surviving rats were subjected to follow‐up echocardiography at 3 months of age (2 weeks after the cessation of ADR treatment) and used for subsequent analysis.

### 2.6 Echocardiography

The echocardiographic inspection was performed using a small animal echocardiography analysis system (Vevo770 and Vevo3100, Canada) as previously described.^20^, ^22^ The parameters of the left ventricular (LV) diameter (LVID) and LV anterior wall (LVAW) at end‐diastole and end‐systole, and LV ejection fraction (LVEF) and LV percentage fractional shortening (LVFS) were measured. The mean value of at least three continuous cardiac cycles was recorded.

### 2.7 Histological analysis

Cardiac tissues were fixed with formaldehyde (4%), mounted in paraffin and then sectioned (3–4 μm in thickness) using a Tissue‐Tek^®^ Sledge microtome (IVS‐410, SAKURA, Japan). The sections were first stained with H&E and then observed under a light microscope (NanoZoomer S60, C13210, Hamamatsu Photonics, Japan) as previously described.^20^, ^21^ All sections were analyzed using the NDP.view2 image viewing software. For the transmission electron microscopy (TEM) analysis, cardiac tissues were routinely fixed in glutaraldehyde (2.5%) and phosphate buffer (0.1 M, pH 7.4), and then fixed in osmium tetroxide buffer (1%) for 1 h. The sections were examined under a JEM‐1230 TEM after uranyl acetate and lead citrate staining as described previously.^18^, ^21^

### 2.8 Protein extraction and immunoblotting

Total protein lysates were prepared from rat heart tissues using protein extraction reagent (USA, Thermo, 78510) as described previously.^21^, ^23^ A mitochondria/cytosol fractionation kit (UK, Abcam, ab65320) was used for isolation of mitochondrial and cytosolic fractions from cardiac tissues, following the instructions in the manual.^24^ Protein extracts were first separated through SDS‐PAGE and then transferred to nitrocellulose membranes. The membranes were incubated at 4℃ overnight with appropriate primary antibodies (ISCA1 [USA, Thermo, PA5‐60121]; TOMM20 [USA, Thermo, PA5‐52843]; NDUFA9 [UK, Abcam, ab14713]; NDUFS3 [UK, Abcam, ab110246]; SDHB [UK, Abcam, ab14714]; GAPDH [UK, Abcam, ab201822]) and subsequently kept for 1 h at RT with the appropriate secondary antibodies. Western blot images were acquired (Bio‐Rad, ChemiDoc XRS+Gel Imaging System, USA) and quantitatively analyzed with Image J software.

### 2.9 Measurement of mitochondrial respiratory complex enzyme activity

Activity analysis of mitochondrial respiratory complexes I, II and IV were performed using enzyme assay kits (UK, Abcam, ab109721, ab109908, ab109911) according to the manufacturer's protocols, as previous described.^25^ In brief, 30 mg of heart tissue from rats were homogenized for harvesting the extracts of mitochondria. Microplate wells were precoated with specific capture antibodies for measuring the activity of complexes I, II and IV. The samples were then added to the microplate wells to capture enzyme activity and incubated. Enzyme activities were measured using a colorimetric method.

### 2.10 ATP measurement

An ATP assay kit (USA, Biovision, K354‐100) was used for measurement of myocardial ATP content as previous described.^26^ Briefly, 40 mg of cardiac tissues from rats were homogenized in 400 µl of ATP assay buffer and deproteinized using a deproteinizing sample preparation kit (UK, Abcam, ab204708). The standard curve was prepared based on the colorimetric assay and the absorbance was assayed in a micro‐plate reader at 570 nm.

### 2.11 Statistical analysis

The experimental data are expressed as means ± SD and analyzed with unpaired two‐tailed Student's *t* tests for two groups or one‐way analysis of variance (ANOVA, with Tukey correction) for multiple groups. GraphPad Prism8 software is used for statistical analysis and *p* < .05 was considered significant.

## 3 RESULTS

### 3.1 Generation of myocardium‐specific *Isca1* knockout heterozygote and observation of basic physiological parameters

The *Isca1* conditional knockout (*Isca1* cKO) rat and the *α‐MHC‐Cre* transgenic rats were established in our lab. Further information can be found in our rat database (www.ratresource.com). The myocardium‐specific Isca1 knockout heterozygote (referred as *Isca1* HET) rats were obtained by crossing *Isca1* cKO rats with the *α‐MHC‐Cre* rats (Figure 1C). Genotyping was carried out by PCR and *Isca1^flox/+^/α‐MHC‐Cre* (with loxp band at 1401 bp and wt band at 1333 bp, and Cre gene positive) rats and WT littermates were used in our subsequent studies (Figure 1D). The protein expression level was confirmed by western blot and ISCA1 protein knockdown efficiency reached 46.3% in myocardial tissues (Figure 1E,F, *n* = 3 rats per group, *p* < .05 vs. WT).

We then performed general observations on rats at 6 months of age and determined several main physiological parameters, including body weight, water and food intake, BP and ECG parameters. The HET rats exhibited no difference compared with the WT rats in these parameters, including systolic pressure, diastolic pressure, mean BP, QRS complex and QTc interval duration (Table 1).

**TABLE 1:** The main physiological parameter of *Isca1* HET rats at 6 months of age

Group Number	WT 10	KO 8
Body weight (g)	452.25 ± 132.08	389.13 ± 48.93
Water intake (ml)	15.88 ± 3.82	16.06 ± 2.65
Food intake (g)	25.09 ± 3.49	26.63 ± 3.37
Systolic pressure (mmHg)	133.60 ± 14.43	127.03 ± 13.55
Diastolic pressure (mmHg)	64.937 ± 15.297	64.080 ± 9.222
Mean blood pressure (mmHg)	88.00 ± 14.56	85.15 ± 9.05
QRS complex (s)	0.047 ± 0.005	0.045 ± 0.005
QTc interval duration (s)	0.080 ± 0.006	0.077 ± 0.005

### 3.2 *Isca1* HET rats exhibited a typical pathological phenotype of DCM

**FIGURE 2:**
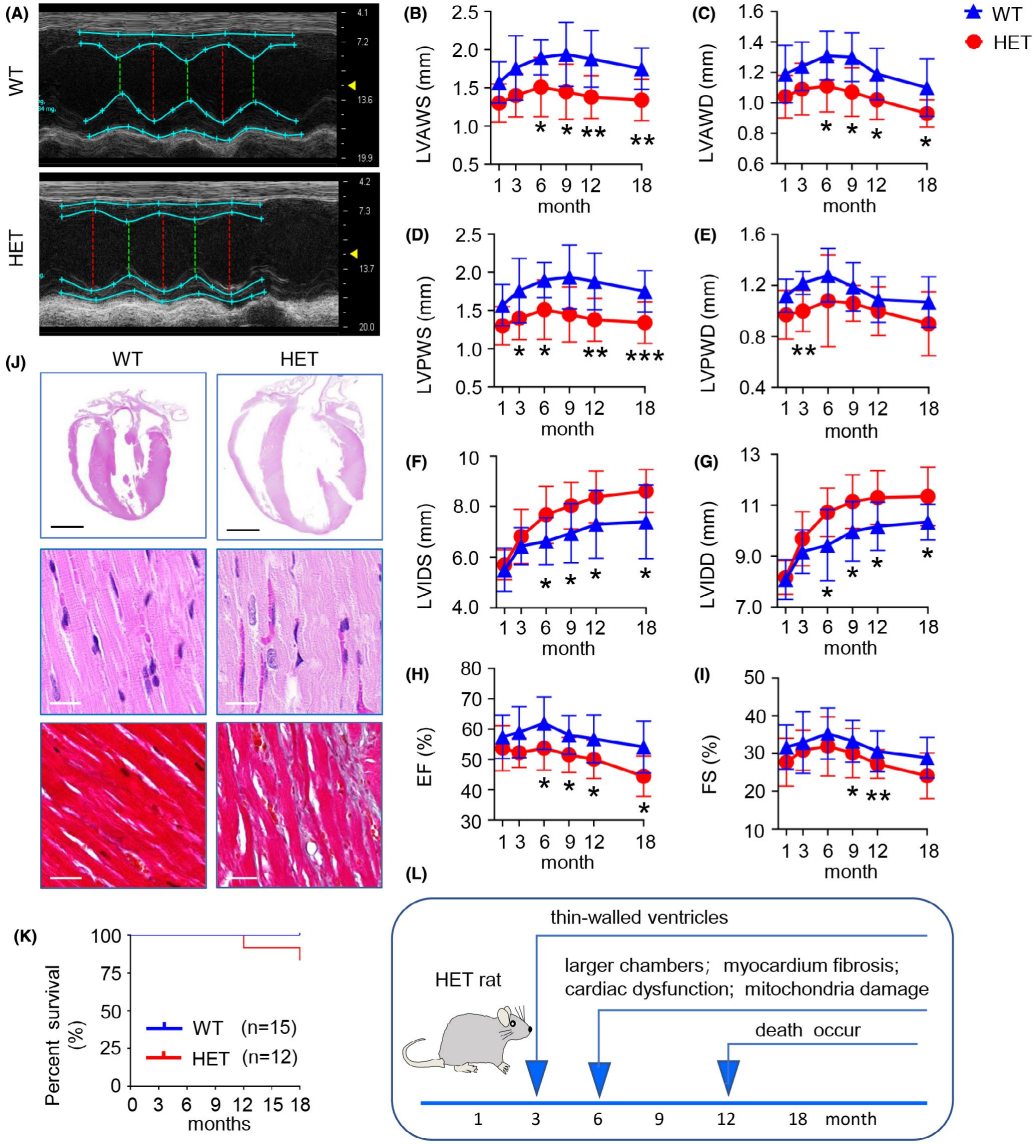
*Isca1* HET rats exhibited a typical pathological phenotype of DCM. (A) The M‐mode echocardiography screenshot of rats at 6 months of age. (B–I) Echocardiographic parameters LVAWS, LVAWD, LVPWS, LVPWD, LVIDS, LVIDD, EF and FS were analyzed at 1, 3, 6, 9, 12, 18 months (*p < .05, **p < .01, ***p < .001, vs. WT). (J) Representative photographs of whole‐heart longitudinal sections with H&E staining (scale bar = 5 mm), magnification of H&E and Masson trichrome‐stained sections of the left ventricle (scale bar = 20 μm) from two groups at 6 months of age. (K) Cumulative percentage survival rate for WT (*n* = 15) and HET (*n* = 12) groups were calculated from 1 to 18 months. (L) Schematic diagram of phenotypic development in HET rats

To study the effects of ISCA1 knockdown expression on geometry and function of heart, we performed echocardiography on HET and WT rats at 1, 3, 5, 7 and 10 months of age. We found that the *Isca1* HET rats exhibited DCM characteristics, including thin‐walled ventricles, larger chambers, and cardiac dysfunction from 3 months of age. This was demonstrated by decreased left ventricle (LV) anterior wall thickness (LVAW) and LV posterior wall thickness (LVPW), increased LV diameter (LVID) both at end‐systole and end‐diastole, and decreased LV ejection fraction (LVEF) and LV fractional shortening (LVFS) (Figure 2A–I, *n* = 10–12 in WT group and *n* = 8–13 in HET group, *p* < .05, *p* < .01 vs. WT).

The morphological phenotypes of DCM in ISCA1 HET rats at 6 months of age were further confirmed by histological observation, including H&E and Masson staining. We found myocardium breakdown and lysis, and myocardium fibrosis in ISCA1 HET rats (Figure 2J).

**FIGURE 3:**
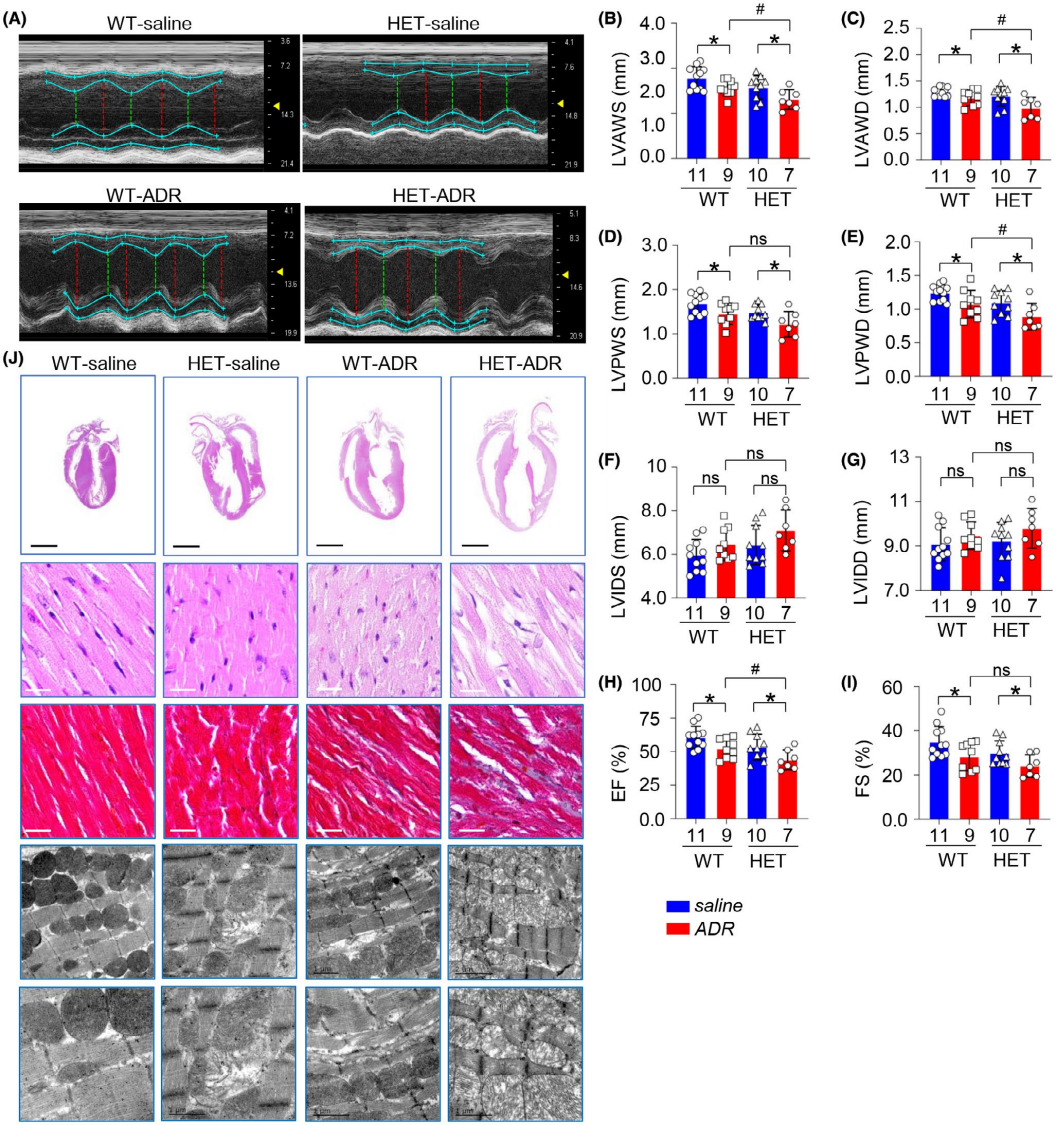
Downregulated Isca1 further impaired cardiac pathological processes at the global and organizational levels. (A) The M‐mode echocardiography screenshot of WT‐saline, WT‐ADR, HET‐saline and HET‐ADR rats at 2 weeks after cessation of ADR treatment. (B–I) Echocardiographic parameters LVAWS, LVAWD, LVPWS, LVPWD, LVIDS, LVIDD, EF and FS for four groups at 2 weeks after cessation of ADR treatment (*p < .05, WT‐saline vs. WT‐ADR or HET‐saline vs. HET‐ADR, ^#^ *p* < .05, HET‐ADR vs. WT‐ADR, ns, no significant). (J) Representative photographs of the whole‐heart longitudinal sections with H&E staining (scale bar = 5 mm), magnification of H&E and Masson trichrome‐stained sections of the left ventricle (scale bar = 20 μm), and TEM analysis of sarcomeres and mitochondria of left ventricular free walls (scale bar = 2 μm) from four groups at 2 weeks after cessation of ADR treatment

We then performed survival rate observations. Cumulative rat mortality data from WT and HET groups were recorded. There were no deaths in the WT group (*n* = 15); however, the survival rate was 83.3% in HET group (*n* = 12) at the end of the observation period (18 months of age) (Figure 3A). The gross anatomy of the dead HET rats revealed dilated ventricles, thin wall thickness and congestion in the ventricles. The characteristics of mitochondrial and metabolic disorders and cardiomyopathy phenotypes in HET rat is summarized in Figure 2L.

### 3.3 Downregulated Isca1 leads to deterioration of cardiac pathological processes at the global and organizational levels

Subsequently, we treated the HET and WT rats with ADR to assess their response to stress.

The WT‐ADR rats exhibited DCM/heart failure (HF) phenotypes induced by ADR treatment, as demonstrated by the decreased LVAW and LVPW both at end‐systole and end‐diastole (Figure 3A–E, *p* < .05 vs. WT‐saline) at the end of observation (2 weeks after cessation of ADR treatment). While LVID exhibited no difference at the end of observation (Figure 3F–G). Cardiac function was also impaired in the WT‐ADR group, as demonstrated by the decreased LVEF and LVFS (Figure 3H–I, *p* < .05 vs. WT‐saline).

We found that ISCA1 knockdown expression exacerbated cardiac geometry disruption and dysfunction under ADR treatment. LVAWS and LVPWS decreased 12.4% and 13.6%, and LVIDS increased 8.5%, respectively, in WT‐ADR group compared with the WT‐saline group; however, those parameters changed to 17.1%, 17.6% and 10.4%, respectively, in the HET‐ADR group compared with the HET‐saline group. LVEF decreased 13.9% in the WT‐ADR group compared with the WT‐saline group, while it decreased 17.63% in the HET‐ADR group compared with the HET‐saline group (Figure 3B–I).

After assessing changes in cardiac geometry and function, pathological changes in response to ADR‐induced stress were further detected in WT and HET groups by H&E and Masson staining and transmission electron microscopy (TEM). The thinning wall, dilated chamber and malalignment in WT‐ADR rats were further damaged by ISCA1 knockdown expression in the HET‐ADR group, consistent with the gross morphological observations obtained by echocardiography. Collagen accumulation in the interstitial space, myocardiolysis and swollen mitochondria were also aggravated in the HET‐ADR group compared with the WT‐ADR group (Figure 3J).

### 3.4 *Isca1* HET rats exhibited complex mitochondrial damage affecting both structure and function in myocardium

**FIGURE 4:**
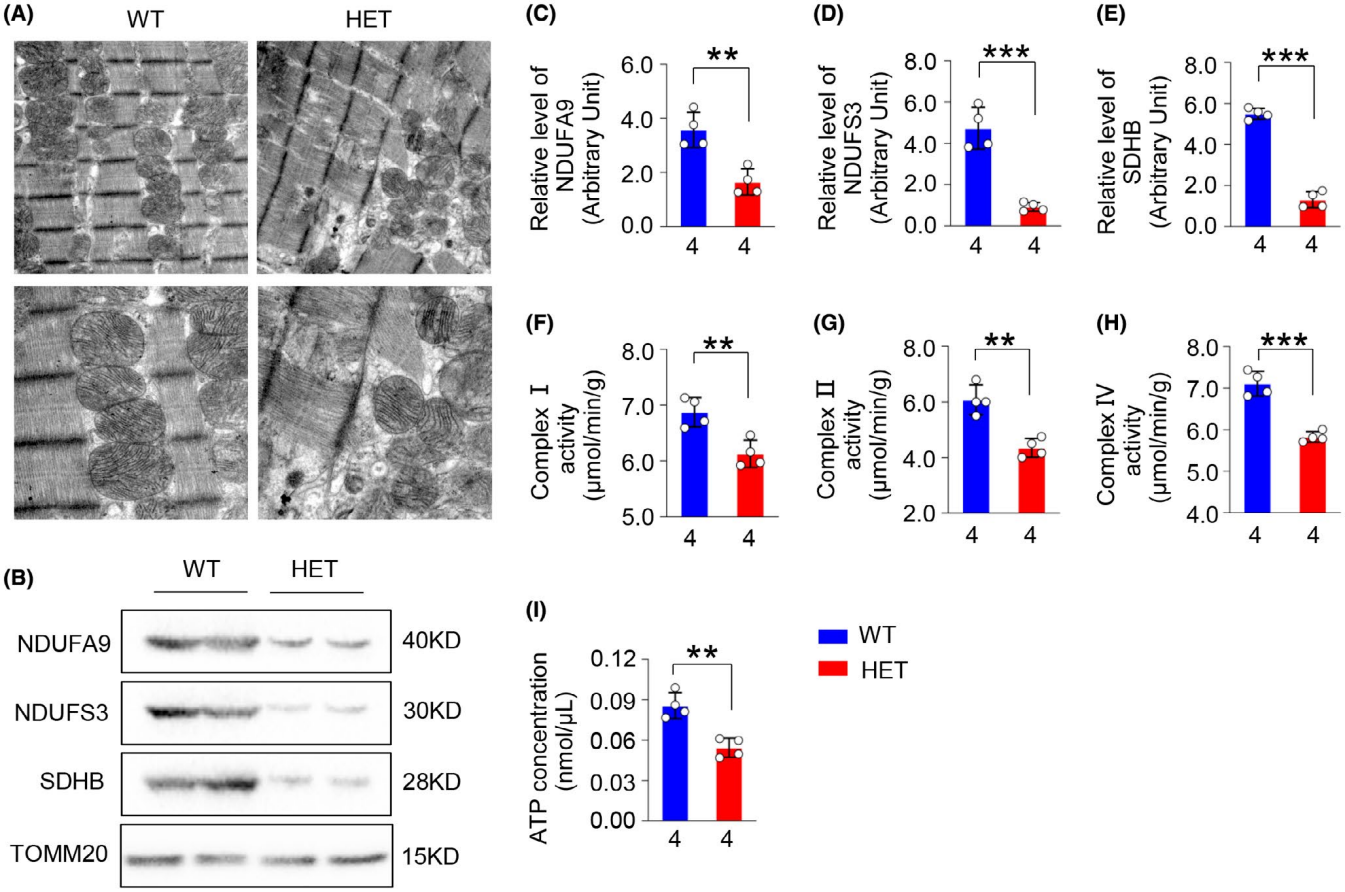
*Isca1* HET rats exhibited complex mitochondrial damage in both structure and function in myocardium. (A) TEM analysis of left ventricular free walls from rats of WT and HET groups at 6 months of age (scale bar = 2 μm). (B–E) NDUFA9, NDUFS3, and SDHB protein expression levels in myocardial mitochondria from two groups at 6 months of age were detected using western blot and quantitative analysis using TOMM20 for normalization (*n* = 4 in WT group and *n* = 4 in HET group, **p < .01, ***p < .001, vs. WT). (F–H) complex Ⅰ, Ⅱ and Ⅳ enzyme activity were detected using a colorimetric method in myocardial mitochondria from WT and HET groups at 6 months of age (*n* = 4 in WT group and *n* = 4 in HET group, **p < .01, ***p < .001, vs. WT). (I) ATP generation was determined in myocardial tissue from WT and HET groups at 6 months of age by colorimetry (*n* = 4 in WT group and *n* = 4 in HET group, **p < .01, vs. WT)

To detect the typical characteristics of MMDS in myocardium from our HET rats, we first detected the mitochondrial morphological changes through TEM. Poorly organized myocardium and myocardiolysis, and swollen mitochondria with damaged membrane structure and partial absence of crests were observed in myocardium from HET rats at 6 months of age (Figure 4A).

We subsequently measured the expression levels of key proteins and enzyme activity for complex Ⅰ, Ⅱ and Ⅳ in HET rats. We found, using western blots, that the mitochondrial complex Ⅰ subunit, including the ubiquinone oxidoreductase subunit A9 (NDUFA9) and the ubiquinone oxidoreductase core subunit S3 (NDUFS3), and the complex Ⅱ subunit succinate dehydrogenase complex iron sulfur subunit B (SDHB), were obviously decreased in myocardium from HET rats (Figure 4B–E, *p* < .01, *p* < .001, vs. WT group). The enzyme activity of complexes Ⅰ, Ⅱ and Ⅳ also decreased significantly in myocardium from HET rats (Figure 4F–H, *p* < .01, *p* < .001, vs. WT group).

We also measured ATP generation, and the concentration decreased significantly in myocardium from HET rats (Figure 4I, *p* < .01, vs. WT group). These data showed that decreased ISCA1 expression severely damaged the mitochondrial complex and impaired energy generation in myocardium.

## 4 DISCUSSION

The heart is highly dependent on mitochondrial metabolism to meet its enormous energy requirements. According to research findings, >40% of the cytoplasmic space in adult cardiac myocytes is occupied by mitochondria.^27^ Maintaining a healthy mitochondrial population is of paramount importance for cardiac homeostasis, since damaged mitochondria produce less ATP and generate dangerous amounts of reactive oxygen species (ROS), ultimately leading to multiple cardiovascular diseases.^28^, ^29^, ^30^, ^31^ Mitochondrial injury is associated with various cardiovascular diseases (CVDs), such as hypertension, atherosclerosis, ischemia‐reperfusion (I/R) injury, metabolic and genetic cardiomyopathies, heart failure and ischemic stroke.^32^, ^33^, ^34^, ^35^, ^36^, ^37^ However, the specific mechanism whereby mitochondrial dysfunction induces cardiac developmental disorders and multiple cardiovascular diseases is not yet well understood, and there is an urgent need for suitable *in vivo* animal models to aid research.

Mutations in the genes involved in synthesis of ISC may severely impair diverse mitochondrial metabolic pathways and interfere with energy production,^3^, ^5^, ^6^ and gene mutations involved in ISC synthesis are closely related to MMDS. Furthermore, abnormal muscle or heart development has been observed in MMDS,^1^, ^9^, ^38^ and among the genes involved in this disease, abnormal ISCA1 has been shown to cause a subtype of the disease, MMDS.^39^ So far, two variants have been reported, p.(Glu87Lys) and p.(Tyr101Cys), and clinical symptoms are reported in patients carrying pathogenic variants of ISCA1, including white matter abnormalities, early onset neurological deterioration, seizures, dilated ventricles, etc.^7^, ^11^, ^12^, ^39^, ^40^, ^41^

Our results demonstrated that our HET model rats exhibited typical pathological phenotypes of DCM, and downregulated ISCA1 exacerbated cardiac pathological process under stress. We also recorded destruction of mitochondrial structure and function in myocardium with ISCA1 deficiency. Therefore, our findings are consistent with the mitochondrial damage and cardiomyopathy phenotypes found in clinical patients with *Isca1* mutations.^7^, ^11^

Myocardium specific *Isca1* knockout heterozygote rats were generated using the *Cre‐lox*P system by crossing *Isca1* cKO rats with *α‐MHC‐Cre* rats. Offspring with a positive *α‐MHC‐Cre* transgene and *Isca1^flox/+^* genotype were selected as myocardium specific *Isca1* knockout heterozygote (*Isca1^flox/+^/α‐MHC‐Cre*) rats. Currently, the knockout heterozygote model exhibits two recognized advantages. First, the homozygous phenotype is severe and death occurs in early childhood. Premature death of the homozygote makes it unsuitable for the study of heart disease and mitochondrial metabolism in adults. However, the longer survival of the heterozygotes is beneficial for carrying out research related to drug efficacy and development. In addition, heterozygous rats are easy to reproduce and are thus more readily available. Therefore, they are more suitable for use in the large‐scale screening and research and development of related drugs.

In this study, our established myocardium specific *Isca1* knockout heterozygote rats exhibited typical pathological phenotypes of DCM and complex mitochondrial damage in both structure and function in myocardium. Therefore, this rat model can be applied to multiple research areas, including abnormal energy metabolism, mitochondrial morphology damage, mitochondrial dysfunction and cardiomyopathy. Consequently, the model can also be used to investigate the pathogenesis of the above related diseases and drug development.

## CONFLICT OF INTEREST

None.

## AUTHOR CONTRIBUTIONS

All listed authors meet the requirements for authorship. LFZ and DL conceived and designed the experiments and wrote the main manuscript text. YHL performed most of the experiments. YWM and XZ contributed to establishment of the animal models and sequence analysis. WC contributed to microinjection technique. WD contributed to measurement of echocardiographic parameters. SG, XG and SP contributed to the animal breeding and management. JXM and FFG contributed to the measurement of physiological parameters. XLQ contributed to the measurement of complex activity.

## Supporting information

Table S1
