# Supplementary material for: Novel rat model of multiple mitochondrial dysfunction syndromes (MMDS) complicated with cardiomyopathy
Source: Animal Model Exp Med. 2021 Dec 6;4(4):381–90. doi: 10.1002/ame2.12193 (PMC8690978; doi:10.1002/ame2.12193)
Supplement: Supplementary file 1 — Table S1 [file AME2-4-381-s001.docx]

**Title:** Novel rat model of multiple mitochondrial dysfunction syndromes (MMDS) complicated with cardiomyopathy

**Running title**: A rat model of MMDS with cardiomyopathy

**Authors:** Yahao Ling^1^, Jiaxin Ma^2^, Xiaolong Qi^2^, Xu Zhang^2^, Qi Kong^2^, Feifei Guan^2^, Wei Dong^1^, Wei Chen^1^, Shan Gao^1^, Xiang Gao^1^, Shuo Pan^2^, Yuanwu Ma^1^, Dan Lu^2*^, Lianfeng Zhang^1*^

1. Key Laboratory of Human Disease Comparative Medicine, National Health Commission of China (NHC), Institute of Laboratory Animal Science, Peking Union Medical College, Chinese Academy of Medical Sciences, China.
2. Beijing Engineering Research Center for Experimental Animal Models of Human Diseases, Institute of Laboratory Animal Science, Peking Union Medical College, Chinese Academy of Medical Sciences, China.

***Corresponding author:**

Lian-Feng Zhang, Ph.D. and Dan Lu, Ph.D.

Building 5, PanjiayuanNanli, Chaoyang District, Beijing 100021 P. R. China.

E-mail: [zhanglf@cnilas.org](mailto:zhanglf@cnilas.org) or [lvd@cnilas.org](mailto:lvd@cnilas.org); Phone and Fax: 86-010-67776394.

**Table 1.** The sequences of primers in this study

| Name | Sequence (5’→3’) | Application |
| --- | --- | --- |
| *Isca1* flox F | ATGGTTCCAGCACTTTGAAGG | Genotyping for  ISCA1 flox |
| *Isca1* flox R | AAGCTAATATGACAGTGGTGAGGC |  |
| Cre F | AACATGCTTCATCGTCGGTC | Genotyping for  transgenic Cre |
| Cre R | GTGCCTTCTCTACACCTGCG |  |
